# Upper extremity deep vein thrombosis associated with systemic lupus erythematosus and antiphospholipid syndrome

**DOI:** 10.1002/jgf2.70023

**Published:** 2025-04-18

**Authors:** Masahiro Yabe, Nozomi Ozaki, Nobuhiro Sato, Norihito Nakamura, Azusa Aoyama, Shuichi Murakami, Yasuo Hirose

**Affiliations:** ^1^ Department of General Internal Medicine Niigata City General Hospital Niigata Japan; ^2^ Department of Laboratory Medicine Niigata City General Hospital Niigata Japan; ^3^ Department of Emergency and Critical Care Medicine Niigata City General Hospital Niigata Japan; ^4^ Department of Cardiology Niigata City General Hospital Niigata Japan; ^5^ Department of Cardiology Tokai University School of Medicine Isehara Japan; ^6^ Department of Neurology Niigata City General Hospital Niigata Japan; ^7^ Department of Nephrology and Rheumatology Niigata City General Hospital Niigata Japan

**Keywords:** antiphospholipid syndrome, intracranial calcifications, neuropsychiatric systemic lupus erythematosus, systemic lupus erythematosus, upper extremity deep vein thrombosis

A 35‐year‐old woman with epilepsy and infertility presented with a 2‐day history of left upper extremity swelling. Symptoms began after traveling, likely due to prolonged immobility followed by vigorous upper extremity activity and pressure from a leather bag on the left arm, contributing to venous stasis. She also reported experiencing recurrent headaches over the past 6 months. The patient was taking levetiracetam, lamotrigine, folic acid, and loxoprofen, none of which are associated with thrombotic risk. On examination, the left upper extremity exhibited swelling (Figure 1A) and superficial venous dilatation extending to the chest (Figure 1B). Laboratory findings demonstrated leukopenia, lymphopenia, a prolonged activated partial thromboplastin time, and a D‐dimer level of 0.9 μ/mL (reference <1.0 μ/mL).

**FIGURE 1:**
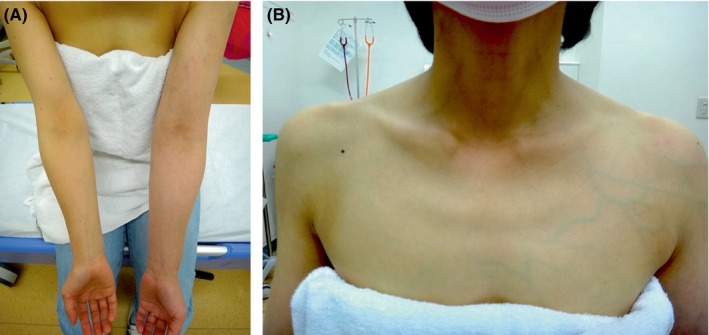
Physical examination demonstrated marked swelling of the left upper extremity (A) and superficial venous dilatation extending from the ipsilateral upper extremity to the upper chest region (B). These findings are consistent with venous congestion secondary to upper extremity deep vein thrombosis.

Chest contrast‐enhanced computed tomography (CT) revealed thrombosis in the left axillary to the subclavian vein (Figure 2A), with stenosis at the clavicle‐first rib junction. Brain CT demonstrated calcifications in the cerebellar dentate nuclei and globus pallidus (Figure 2B). Serologic markers (antinuclear, anti‐dsDNA, anti‐β2 glycoprotein I, anticardiolipin) and hypocomplementemia confirmed systemic lupus erythematosus (SLE) and antiphospholipid antibody syndrome (APS). The patient fulfilled the 2012 Systemic Lupus International Collaborating Clinics classification criteria, 2019 European League Against Rheumatism/American College of Rheumatology Classification Criteria for SLE, and 2006 revised Sapporo classification criteria for APS, confirmed upper extremity deep vein thrombosis (UEDVT), APS, and SLE.

**FIGURE 2:**
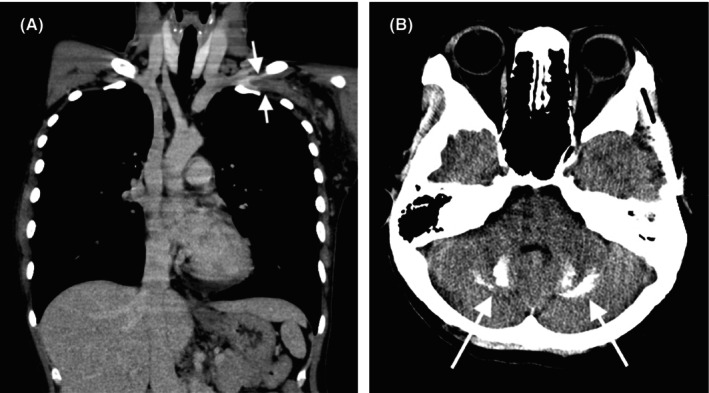
Contrast‐enhanced computed tomography of the best revealed thrombosis extending from the left axillary to the subclavian vein (A, arrow), with significant venous obstruction. Concurrently, plain computed tomography of the brain identified calcifications with the bilateral cerebellar dentate nuclei and globus pallidus (B, arrow), hallmark features suggestive of neuropsychiatric systemic lupus erythematosus.

Percutaneous balloon angioplasty and aspiration thrombectomy were performed, followed by anticoagulation with warfarin. Corticosteroids, mizoribine, and hydroxychloroquine were initiated for SLE‐associated leukopenia, resulting in clinical improvement without symptom recurrence.

UEDVT accounts for ~10% of DVT cases in Western countries but only 3% in Japan.^1^, ^2^ Common symptoms include upper extremity discomfort, swelling, and superficial venous dilatation.^1^ UEDVT is classified as primary (e.g., venous thoracic outlet syndrome and Paget‐Schroetter syndrome) or secondary (e.g., central venous catheters, malignancy, trauma, pregnancy, and oral contraceptive use).^1^ In this case, extrinsic compression of the left subclavian vein at the costoclavicular junction, combined with prolonged immobility followed by vigorous upper extremity activity, contributed to venous thoracic outlet syndrome and Paget‐Schroetter syndrome, which increased the risk of UEDVT.

D‐dimer measurement is a valuable diagnostic tool for suspected UEDVT. In this patient, D‐dimer (0.9 μ/mL) was within institutional reference limits but mildly elevated under Western cut‐offs (0.5 μ/mL). When the pre‐test probability of venous thromboembolism is high, the negative predictive value of D‐dimer decreases, increasing the risk of false‐negative results. Imaging is recommended in such cases.^3^

Although rare, UEDVT may be the initial manifestation of APS or SLE. APS, a common complication of SLE, presents with venous or arterial thrombosis, particularly in young women with a history of infertility.^4^ Neuropsychiatric SLE (NPSLE) is classified into central and peripheral types. Central NPSLE can present as focal symptoms, such as headaches (28.3%), epilepsy (20.0%), and cerebrovascular disease (15.0%), and imaging findings such as basal ganglia and cerebellar calcifications are hallmark findings of this condition.^5^ UEDVT should be suspected in patients with ipsilateral upper extremity swelling and superficial venous dilatation. In this case, the coexistence of UEDVT and intracranial calcifications strongly supports the diagnosis of APS in SLE.

## AUTHOR CONTRIBUTIONS

**Masahiro Yabe:** Conceptualization; writing – original draft; methodology; project administration; data curation; investigation. **Nozomi Ozaki:** Conceptualization; writing – original draft; data curation; investigation. **Nobuhiro Sato:** Supervision; writing – review and editing. **Norihito Nakamura:** Supervision; writing – review and editing. **Azusa Aoyama:** Supervision; writing – review and editing. **Shuichi Murakami:** Supervision; writing – review and editing. **Yasuo Hirose:** Supervision; writing – review and editing.

## CONFLICT OF INTEREST STATEMENT

The authors declare no conflicts of interest related to this article.

## ETHICS STATEMENT

Not applicable.

## PATIENT CONSENT

Written consent to publish this report was obtained from the patient.
